# Is Umbilical Cord Blood Therapy an Effective Treatment for Early Lung Injury in Growth Restriction?

**DOI:** 10.3389/fendo.2020.00086

**Published:** 2020-03-03

**Authors:** Beth J. Allison, Hannah Youn, Atul Malhotra, Courtney A. McDonald, Margie Castillo-Melendez, Yen Pham, Amy E. Sutherland, Graham Jenkin, Graeme R. Polglase, Suzanne L. Miller

**Affiliations:** ^1^The Ritchie Centre, Hudson Institute of Medical Research, Clayton, VIC, Australia; ^2^Department of Obstetrics and Gynaecology and Paediatrics, Monash University, Clayton, VIC, Australia; ^3^Monash Newborn, Monash Medical Centre, Clayton, VIC, Australia

**Keywords:** growth restriction, ventilation induced lung injury, umbilical cord blood (UCB), treatment, animal model

## Abstract

Fetal growth restriction (FGR) and prematurity are often co-morbidities, and both are risk factors for lung disease. Despite advances in early delivery combined with supportive ventilation, rates of ventilation-induced lung injury (VILI) remain high. There are currently no protective treatments or interventions available that target lung morbidities associated with FGR preterm infants. Stem cell therapy, such as umbilical cord blood (UCB) cell administration, demonstrates an ability to attenuate inflammation and injury associated with VILI in preterm appropriately grown animals. However, no studies have looked at the effects of stem cell therapy in growth restricted newborns. We aimed to determine if UCB treatment could attenuate acute inflammation in the first 24 h of ventilation, comparing effects in lambs born preterm following FGR with those born preterm but appropriately grown (AG). Placental insufficiency (FGR) was induced by single umbilical artery ligation in twin-bearing ewes at 88 days gestation, with twins used as control (appropriately grown, AG). Lambs were delivered preterm at ~126 days gestation (term is 150 days) and randomized to either immediate euthanasia (unventilated controls, AG_UVC_ and FGR_UVC_) or commenced on 24 h of gentle supportive ventilation (AG_V_ and FGR_V_) with additional cohorts receiving UCB treatment at 1 h (AG_CELLS_, FGR_CELLS_). Lungs were collected at post-mortem for histological and biochemical examination. Ventilation caused lung injury in AG lambs, as indicated by decreased septal crests and elastin density, as well as increased inflammation. Lung injury in AG lambs was attenuated with UCB therapy. Ventilated FGR lambs also sustained lung injury, albeit with different indices compared to AG lambs; in FGR, ventilation reduced septal crest density, reduced alpha smooth muscle actin density and reduced cell proliferation. UCB treatment in ventilated FGR lambs further decreased septal crest density and increased collagen deposition, however, it increased angiogenesis as evidenced by increased vascular endothelial growth factor (VEGF) expression and vessel density. This is the first time that a cell therapy has been investigated in the lungs of growth restricted animals. We show that the uterine environment can alter the response to both secondary stress (ventilation) and therapy (UCB). This study highlights the need for further research on the potential impact of novel therapies on a growth restricted offspring.

## Introduction

Fetal growth restriction (FGR) is a common complication of pregnancy, where a fetus fails to reach its expected growth potential, primarily due to placental insufficiency (1). FGR significantly increases the risk of morbidity and pulmonary conditions following preterm birth, with increased rates of bronchopulmonary dysplasia and pulmonary hypertension (2, 3). Despite the increased risk of pulmonary complications, lung pathology following FGR remains contentious. We and others have found comparable lung weight, structure, surfactant protein expression, and ventilation requirements compared to appropriately grown (AG) cohorts (4, 5). However, it is evident that early and late onset FGR have differential effects (6), and animal studies to date have primarily induced FGR during late gestation, and it is, thus, possible that crucial lung development has already occurred at this stage (7); whereas preclinical studies of long term growth restriction describe altered surfactant protein (8, 9) disrupted alveolarization, with thickened parenchyma (10) and large alveoli resulting in reduced alveolar and vascular density (11).

There is currently no cure or therapy for FGR. Current treatment of FGR primarily involves the adjustment of the delivery time, thus infants are often delivered preterm (<37 weeks gestation), particularly those with early-onset FGR (12). Prematurity itself is a significant risk factor for pulmonary morbidity and necessitates medical interventions such as mechanical ventilation. Whilst ventilation is usually essential for survival in such scenarios, it has the potential to exacerbate pathology in FGR lungs, particularly since lung development may already be adversely affected by the chronic hypoxemia caused by placental insufficiency (11). The resultant lung injury after birth is known as ventilation induced lung injury (VILI). VILI and elevated inflammation cause direct tissue injury and in turn, exacerbate lung inflammation. Long term ventilation can reduce alveoli number, disrupt vasculature and alveolar architecture (13–16), hampering lung mechanics and necessitating the further need for assisted ventilation. Current treatment focuses on ensuring the survival of the FGR infant while ameliorating the detrimental effects of FGR on the lungs.

Umbilical cord blood (UCB) cells have been highlighted as a potential treatment for infants born preterm, due to their potent anti-inflammatory properties and easy access (17). Preclinical studies using specific stem like cell populations present within UCB have shown promising anti-inflammatory and immune modulatory effects in VILI of preterm animals (15, 18). UCB provides a unique source of the functionally important stem like cells that may each play a modulatory role in preventing injury (19). UCB therapy has been examined for prevention and repair of brain injury (20, 21), and has also shown promise in clinical trials where administration improved motor and neurodevelopmental outcomes in children with cerebral palsy (22, 23). UCB is comprised of many cell types including cells that mediate hematopoiesis and vascular growth (24–27). UCB also show strong anti-inflammatory benefits, and they are a feasible postnatal treatment with low immunogenicity (25, 26, 28). Within the lung, UCB therapy is thought to reduce inflammation through paracrine effects. Accordingly, we used our established model of ovine fetal growth restriction to examine (i) the effects of preterm birth and ventilation on FGR lungs, and (ii) if UCB could be a potential new treatment for VILI in FGR and/or AG infants. To focus on acute inflammation and injury, this study examined the first 24 h postnatally in FGR and AG preterm lambs.

## Methodology

### Umbilical Cord Blood Collection

Umbilical cord blood (UCB) was collected from separate healthy term ovine pregnancies. UCB was collected during cesarean section under general anesthesia. Approximately 90 mL of UCB was collected from the umbilical veins into heparinized tubes. UCB was diluted 1:1 with phosphate buffered saline and centrifuged at 3,200 rpm at RT for 12 min with no brake. The buffy coat was isolated to obtain the mononuclear cells (MNCs) and red blood cell lysis of this fraction performed. Cells were counted using trypan blue exclusion and a hemocytometer, and cells were cryopreserved at ~25 million cells/ml in freeze media (10% DMSO, 40% FBS and 50% DMEM/F12) until required. A minimum of three cryopreserved UCB donors was pooled after thawing and before administration to reduce intra-sample UCB variation.

### Fetal Surgery

Aseptic surgery was performed on anesthetized (sodium thiopentone 20 mL; Pentothal; Boehringer Ingelheim, Australia; maintenance inhaled isoflurane 2–5%) Border-Leicester pregnant ewes (*n* = 17) carrying twin-pregnancies at 88 days gestation (term is 150 days). Prophylactic antibiotics were administered via the maternal jugular vein, including 5 mL of Engemycin (Engemycin 100, Coopers, MSD Animal Health, New Zealand) and 1 g of ampicillin (Ampicyn 1 g, Mylan N.V., USA). Following a thorough cleaning of the surgical sites, the fetus was exposed via cesarean section. Marcain (5 mL, Marcain (0.5%) with Adrenaline, Aspen Pharmacare Australia NSW, Australia) was applied to all surgical sites prior to incision to provide analgesic coverage. Single umbilical artery ligation was performed by placing two silk ties tightly around one of the umbilical arteries, that causes chronic placental insufficiency and fetal growth restriction (FGR). In control twins, the umbilical cord was handled but not ligated. The fetus was returned to the uterus and abdominal incisions were repaired. A maternal jugular vein catheter was inserted for antibiotic administration. Following surgery ewes were randomly allocated to an experimental group (UVC *n* = 6 ewes, V *n* = 5 ewes or CELLS *n* = 6 ewes).

For 3 consecutive days after surgery, antibiotics [to the fetus (Ampicillin, 1 g via the amniotic catheter] and the ewe [Engemycin 5 mL intravenous (i.v.)] and analgesia [Panadol (100 mg/mL, Apotex, NSW, Australia) suppository] were administered.

### Experimental Design

The ewe and fetuses were monitored daily until 124 days of fetal gestation. At 124 and 125 days, ewes received 11.4 mg betamethasone intramuscularly (Celestone Chronodose, Schering Plow, Sydney, Australia). At 126 days, ewes (*n* = 11) and their fetuses (*n* = 21) in the ventilation groups (AG_V_ *n* = 6, FGR_V_ *n* = 6 and AG_CELLS_ *n* = 6, FGR_CELLS_, *n* = 5) underwent an additional cesarean section or post-mortem (AG_UVC_ *n* = 6, FGR_UVC_ *n* = 5). At this time, there had been *n* = 2 *in utero* deaths in the FGR groups; *n* = 1 in the FGR_UVC_ group and *n* = 1 in the FGR_CELLS_ group, hence the reduced number in these two groups at this timepoint. Following maternal anesthesia (sodium thiopentone 20 mL; maintenance inhaled isoflurane 2–5%), the first lamb was exteriorized and intubated with an endotracheal tube (size 4.0 mm). Lung liquid was drained passively and a transcutaneous arterial oxygen saturation (SpO_2_) probe (Masimo, Radical 4, CA, USA) was placed around the right forelimb of the lamb and the output digitally recorded.

The umbilical cord was then clamped and cut, the lambs were delivered, dried, weighed and placed on an infant warmer (Fisher and Paykel Healthcare, Auckland, New Zealand) for initiation of assisted ventilation. An umbilical vein and artery were immediately catheterized for maintenance of anesthesia and analgesia (Alfaxane i.v. 5 mg/kg/h; Jurox, East Tamaki, Auckland, New Zealand). Arterial pressure was digitally recorded in real-time (1 kHz, Powerlab; ADInstruments, Castle Hill, NSW, Australia). The lambs were anesthetized for the entirety of the experiment to prevent spontaneous breathing. Ventilation was commenced using positive pressure ventilation with PIP set at 30 cmH_2_O and PEEP at 5 cmH_2_O (Babylog 8000+, Dräger, Lübeck, Germany): inspiratory time was 0.4 s and expiratory time was 0.6 s. Lambs were ventilated with warmed, humidified gas with an initial fraction of inspired oxygen (F_i_O_2_) of 0.4 and subsequently adjusted to maintain SaO_2_ between 90 and 95%. At 10 min, all lambs received surfactant (Curosurf, 100 mg/kg, Chiesi Farmaceutica, Italy). At 20 min, ventilation continued in volume guarantee mode set at 5–7 ml/kg, which is the tidal volume for lambs at this gestation (29). Physiological parameters pH and PaCO_2_ were kept within normal limits (7.2–7.4 and 45–55 mmHg, respectively) by adjusting the ventilator rate and inspired O_2_ levels. Lamb well-being was monitored throughout ventilation via assessment of the partial pressure of arterial oxygen (PaO_2_) and carbon dioxide (PaCO_2_), oxygen saturation (SaO_2_), pH, hematocrit, glucose and lacate with regular blood gas samples (ABL 700 blood gas analyzer; Radiometer, Copenhagen, Denmark). Lambs were ventilated for 24 h.

For groups that received UCB (AG_CELLS_ *n* = 6 and FGR_CELLS_ *n* = 5), 25 million cells/kg was administered intravenously to lambs at 1 h after birth, control groups (AG_V_ *n* = 5 and FGR_V_ *n* = 5) were administered the equivalent volume of saline. UCB cells were quantified via cell counts of UCB mononuclear cells to establish an accurate dose prior to administration.

### Post-mortem

At 24 h, ventilated lambs were euthanized with an overdose of 20 mL of phenobarbitone, whilst unventilated control groups were immediately euthanized at 125 ± 1 days gestation via an overdose of phenobarbitone. Lambs were weighed and lungs isolated for collection. The left bronchus was ligated before the left lung was removed distal to the ligature. The left lung was snap frozen in liquid nitrogen for RNA processing. The right whole lung was pressure fixed at 20 cmH_2_O via the trachea with formalin. Nine sections (2 cm^3^) of the lung were randomly selected from an area devoid of major airways from each lobe (upper, middle, lower) and processed for assessment of lung histology. Lung sections were embedded in paraffin, then cut into 5 μm sections and mounted on to Superfrost Plus slides for histological and immunohistochemical analysis.

### Detecting Stem Cell Migration

To detect if UCB stem cells were present in the lungs of treated lambs, the UCB cells were tagged with carboxyfluorescein succinimidyl ester (CFSE) before administration (30). Cut lung sections were dewaxed and counter-stained in Hoechst (Invitrogen, USA) and coverslipped. Stem cell identification was conducted using fluorescent microscopy (Olympus BX-41, Japan).

### Histological Analysis of Lung Morphology

Gross histological pathology and parenchymal elastin was detected via Hematoxylin and Eosin and Hart's elastin stains, respectively, as previously described (31) Masson's Trichrome was used to identify collagen fibers (32). Three sections (obtained as described above) were randomly selected for each histological assessment. Quantification of histology is outlined below.

### Immunohistochemistry

Lung tissue was immunostained for Ki67, α-smooth muscle actin and CD45. Ki67 and α-smooth muscle actin immunostaining was carried out as previously described [(5, 33) see Supplementary Table 1]. For immunostaining of CD45, slides were heated in a 60°C oven for 2 h to remove excess wax, followed with histolene clearing and ethanol rehydrating steps. Antigen retrieval was performed by boiling tissue sections in 0.01 M Citrate buffer (pH 6.0) for 3 × 10 min bursts. Sections were then washed in phosphate buffer solution (PBS) before endogenous peroxidase in the tissue was blocked with 3% hydrogen peroxide for 10 min. Tissue sections were washed and then slides were blocked in Serum-Free Protein Block (DAKO) before incubation with the primary antibody CD45 (BD Pharmingen Rat Anti-Mouse, 1:500) for 60 min. Sections were washed in PBS and then incubated with biotinylated secondary antibody (Rabbit anti-mouse, 1:200) for 60 min followed with streptavidin horseradish peroxidase and developed with diaminobenzidine (DAB) and hydrogen peroxide. Sections were counterstained with hematoxylin and dehydrated with ethanol and histolene before mounting with a coverslip. All immunostains were performed in the presence of a negative control.

### Cytokine Array

To assess cytokine expression in the lungs, frozen lung tissue was weighed out in 50–100 mg quantities, for protein expression of pro-inflammatory and anti-inflammatory cytokines. The concentrations of *interferon gamma (IFN*γ*), interleukin (IL)-17A, IL-21, IL-8, IL-10 TNF*α, and *VEGF-A* in lung tissue lysate were measured using an ovine cytokine array (ovine QAO-CYT-1-1, RayBiotech, Georgia, USA).

### Analysis

For histological and immunohistochemistry analysis, five random fields of view were taken of each section and analyzed by a single blinded observer (H.Y.). Images were non-overlapping and excluded large airways or vessels.

Lung morphology was assessed through quantification of tissue to airspace ratio and density of secondary septal crests as previously described (4). Elastin, collagen and αSMA density were assessed through Smart Segmentation on Image Pro Premier (Media Cybernetics, USA) (16). Elastin and collagen were then expressed as ratios of lung tissue. Manual point counting was utilized to assess Ki67 and CD45 to tissue ratios (16). Measurement of vascular vessel number was assessed using αSMA immunostained tissue by a single observer blinded to the experimental group (B.J.A.). Vessels were identified by positive staining and were only included when a full cross section of the vessel was visible in the field of view.

Data are expressed as mean ± standard error of the mean (SEM). Statistical analysis was performed with SPSS using a mixed model using growth and treatment as factors in all immunohistochemical and morphological assessments and growth, treatment and time in ventilation parameters. Where significant interactions were detected, differences were isolated with *post-hoc* Tukey's testing. Statistical significance was accepted as *P* < 0.05.

## Results

### Lamb Characteristics

Lamb characteristics are presented in Table 1. Single umbilical artery ligation (SUAL) resulted in ~30% overall reduction in birth weight in FGR lambs. SUAL also resulted in the death of two fetuses, one in the UVC and one in the CELLS group. FGR_UVC_ weighed 37% < AG_UVC_ (*P* = 0.0001), whilst FGR_V_ weights were 23% lower than AG_V_ (*P* = 0.04) and FGR_CELLS_ lambs weighed 32% < AG_CELLS_ (*P* = 0.01, Table 1). Lung weight, corrected for body weight, was not different across groups.

**Table 1:** Total sample size, animal and lung weights and sex of fetuses and lambs.

	**AG_**UVC**_**	**FGR_**UVC**_**	**AG_**V**_**	**FGR_**V**_**	**AG_**CELLS**_**	**FGR_**CELLS**_**
n	6	5	6	6	6	5
Animal weight (kg)	3.56 ± 0.13	2.25 ± 0.17^#^	3.21 ± 0.17	2.46 ± 0.22^#^	3.40 ± 0.18	2.29 ± 0.17^#^
Lung corrected for body weight (g/kg)	35.94 ± 1.98	31.04 ± 3.07	29.33 ± 0.91	30.36 ± 3.22	27.32 ± 1.55	29.44 ± 1.90
Males, n (%)	5 (83)	2 (40)	2 (33.3)	2 (33.3)	5 (83)	2 (40)

*AG, appropriately grown; UVC, unventilated control; FGR, growth restricted; V, ventilated; CELLS, animals treated with umbilical cord blood cells*.

# *Indicates p < 0.05 for growth effects using a 2-way ANOVA*.

### Ventilation Parameters

**Figure 1:**
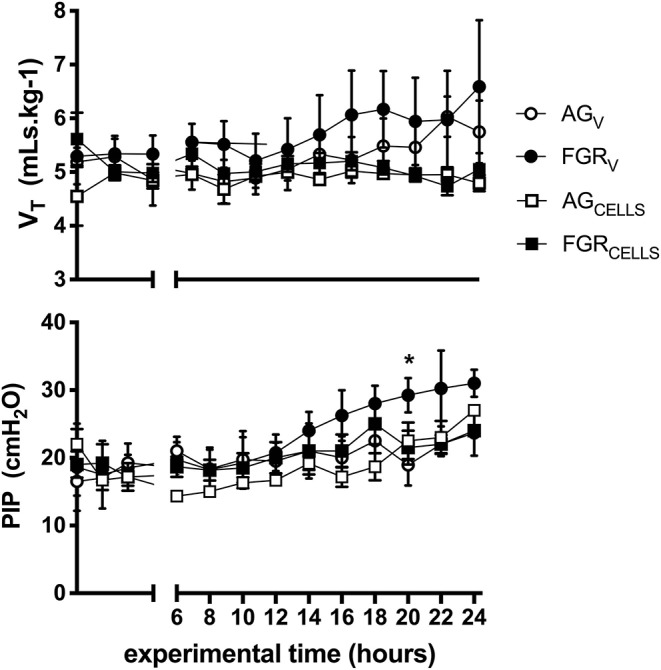
Ventilation parameters. Mean ± SEM tidal volume V_T_ (mLs.kg^−1^) and peak inspiratory pressure (PIP) in appropriately grown (AG_V_, white circles, *n* = 5), growth restricted (FGR_V_, black circles *n* = 5) and appropriately grown and growth restricted treated with umbilical cord blood cells (AG_CELLS_, white squares *n* = 6; FGR_CELLS_, black squares *n* = 5) over the experimental period (hours) ^*^indicates significant differences (*p* < 0.05) across time.

There were no differences in tidal volumes (V_T_, 5–6 mL/kg) between groups. Peak inspiratory pressure (PIP) required to achieve V_T_ was initially not different between groups, however PIPs were significantly (*P* > 0.003) increased at 20 h in FGR_V_ lambs compared to all groups (Figure 1). There was no effect of FGR or UCB on the requirement for PIP. Lung compliance was not different between groups (data not shown).

### Stem Cell Migration

**Figure 2:**
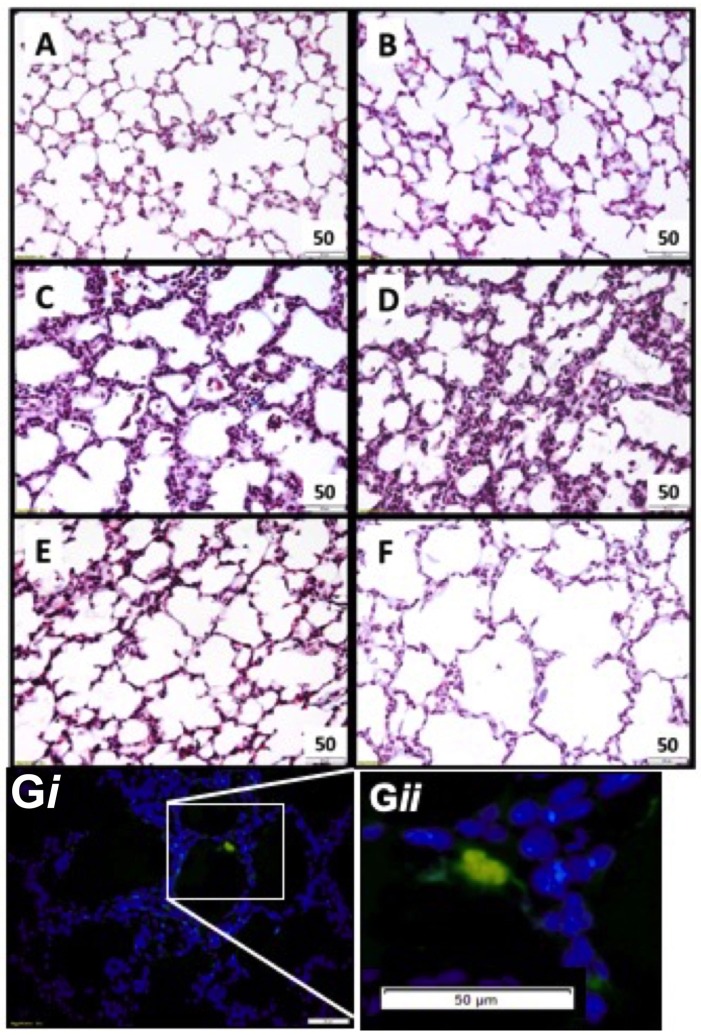
Representative lung morphology images. Photomicrographs of Masson Trichrome stained sections in appropriately grown (**A** AG_UVC_, **C** AG_VENT_, **E** AG_CELLS_) and growth restricted (**B** FGR_UVC_, **D** FGR_VENT_, **F** FGR_CELLS_) animals. Fluorescent tagged cell in lung parenchyma UCB cells present within parenchymal lung tissue (blue) and a fluorescing UCB cell (green). Magnification ×400 (**G***i* and at higher magnification **G***ii*).

Lung tissue was examined for the presence of CFSE tagged UCB cells in all ventilated groups (Figure 2). Fluorescing cells were apparent in all cell treated animals, and were not present in saline controls (Figure 2G).

### Lung Morphology

Two lungs were not appropriately fixed (one from FGR_UVC_ and one from FGR_CELLS_) and were thus excluded from morphological and immunohistochemical analysis. Final sample size for morphological and immunohistochemical analysis is AG_UVC_ *n* = 6, AG_V_ *n* = 6, AG_CELLS_ *n* = 6, FGR_UVC_ *n* = 4, FGR_V_ *n* = 6 and FGR_CELLS_ *n* = 4.

**Figure 3:**
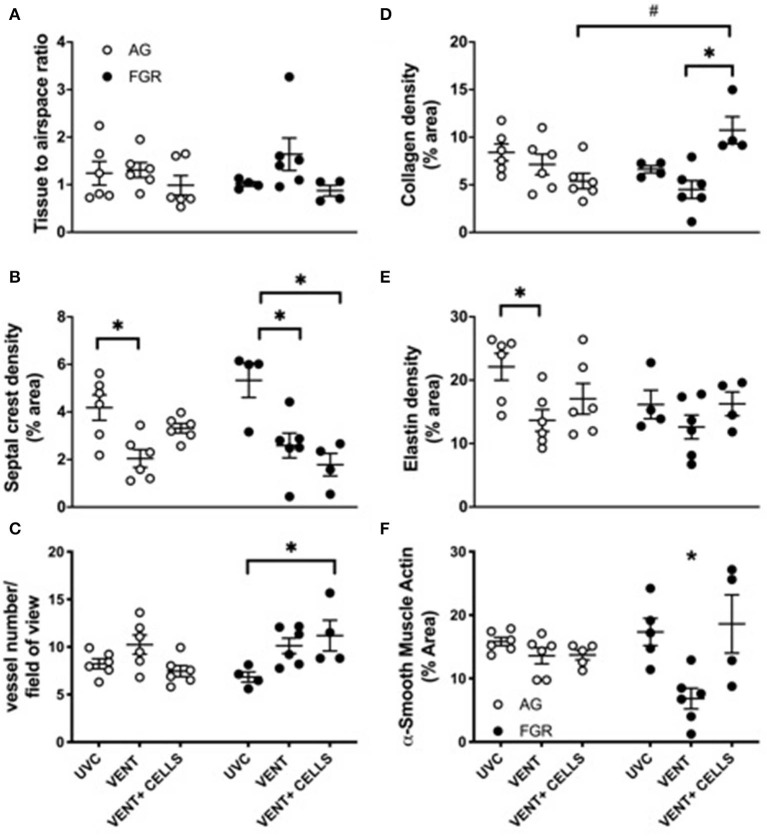
Lung parenchymal and vascular structure. Data are mean ± SEM tissue airspace ratio **(A)**, secondary crest density **(B)**, arteriolar vessel wall number **(C)** and collagen **(D)** elastin **(E)** and α-smooth muscle actin **(F)** density (corrected for total tissue area) in appropriately grown (AG) and growth restricted (FGR) unventilated controls (AG_UVC_ and FGR_UVC_, white circles), following ventilation (AG_VENT_ and FGR_VENT_, black circles) and following ventilation and cell treatment (AG_CELLS_ and FGR_CELLS_, gray circles). Data were compared using a two-way ANOVA. ^*^Indicates *p* < 0.05 treatment effects and ^#^indicates *p* < 0.05 growth effects using a two-way ANOVA.

Tissue to airspace ratio was not altered by FGR or ventilation (Figure 3A). Heterogeneous lung injury was observed in AG_V_ and FGR_V_ compared to their unventilated cohorts, with areas containing thickened blood-air barriers, contrasting against other regions showing prominent airway enlargement. This heterogeneity was reduced with UCB (AG_CELLS_ and FGR_CELLS_), although alveoli remained enlarged (Figure 2). Septal crest density was significantly reduced following ventilation compared to unventilated controls (Figure 3B) resulting in a 57.6% reduction in AG lambs (AG_UVC_ 4.2 ± 0.5 vs. AG_V_ 2.0 ± 0.4, *P* = 0.0001) and 44.6% reduction in FGR lambs (FGR_UVC_ 5.3 ± 0.7 vs. FGR_V_ 2.5 ± 0.5, *P* = 0.002, Figure 3B). Treatment with UCB restored septal crest density in AG (AG_CELLS_ 3.3 ± 0.2, *P* = 0.02) but not FGR lambs.

Vessel number, as assessed in α-smooth muscle actin-stained lungs (Figure 3C). was significantly increased in ventilated growth restricted lambs treated with UCB compared to unventilated, growth restricted lambs (FGR_CELLS_ 11.2 ± 1.6 vs. FGR_UVC_ 6.8 ± 0.5, *P* = 0.04). No differences in vessel density were observed across groups in the AG lambs.

Collagen density was not altered in either AG or FGR lambs following ventilation (Figure 3D). Treatment with UCB significantly increased collagen density in FGR_CELLS_ animals compared to FGR_V_ and AG_CELLS_ lambs (FGR_CELLS_ 10.7 ± 1.4% vs. AG_CELLS_ 5.3 ± 0.8% and FGR_V_ 4.5 ± 0.9%; *p* > 0.02).

Elastin density was significantly reduced in ventilated AG lambs compared to AG control lambs (AG_UVC_ 22.1 ± 2.2% vs. AG_V_ 13.6 ± 1.7%, *P* < 0.05). Elastin density was restored following treatment with UCB in AG lambs (Figure 3E). Elastin density was not different in FGR lambs either with ventilation or UCB treatment.

We assessed positively stained α-smooth muscle actin tissue to determine density (Figure 3F). Ventilation of FGR lambs significantly reduced α-smooth muscle actin density (FGR_V_ 6.8 ± 1.3% vs. FGR_UVC_ 17.3 ± 2.1%). Treatment with UCB reversed this finding (FGR_V_ 6.8 ± 1.3% vs. FGR_CELLS_ 18.6 ± 4.6%). α-smooth muscle actin density was not different in AG lambs either with ventilation or UCB treatment.

### Cell Proliferation

**Figure 4:**
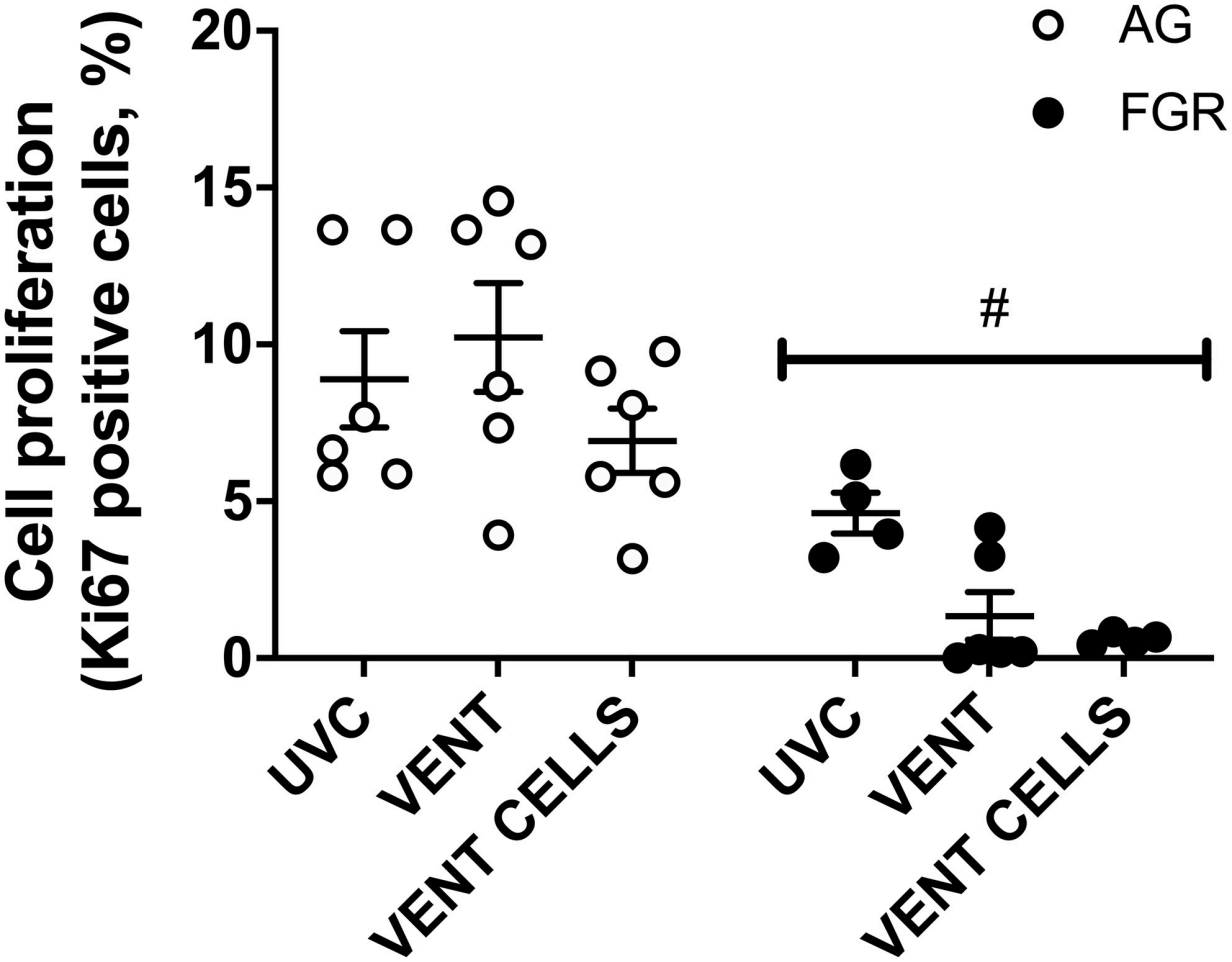
Cell proliferation. Data are mean ± SEM Ki67 (cell proliferation marker) positive cells in appropriately grown (AG, white) and growth restricted (FGR, black) unventilated controls (AG_UVC_ and FGR_UVC_), following ventilation (AG_VENT_ and FGR_VENT_) and following ventilation and cell treatment (AG_CELLS_ and FGR_CELLS_). Data were compared using a two-way ANOVA. ^#^Indicates *p* < 0.05 treatment effects using a two-way ANOVA.

Cell proliferation was assessed in lung parenchyma using the proliferation marker Ki67. Cell proliferation was significantly decreased (*P* < 0.0001, Figure 4) in FGR, compared to AG groups. Cell proliferation was significantly reduced by 80% in FGR_VENT_ compared to AG_VENT_, and also decreased in FGR_CELLS_ compared to AG_CELLS_ (by 90%).

### Inflammation

**Figure 5:**
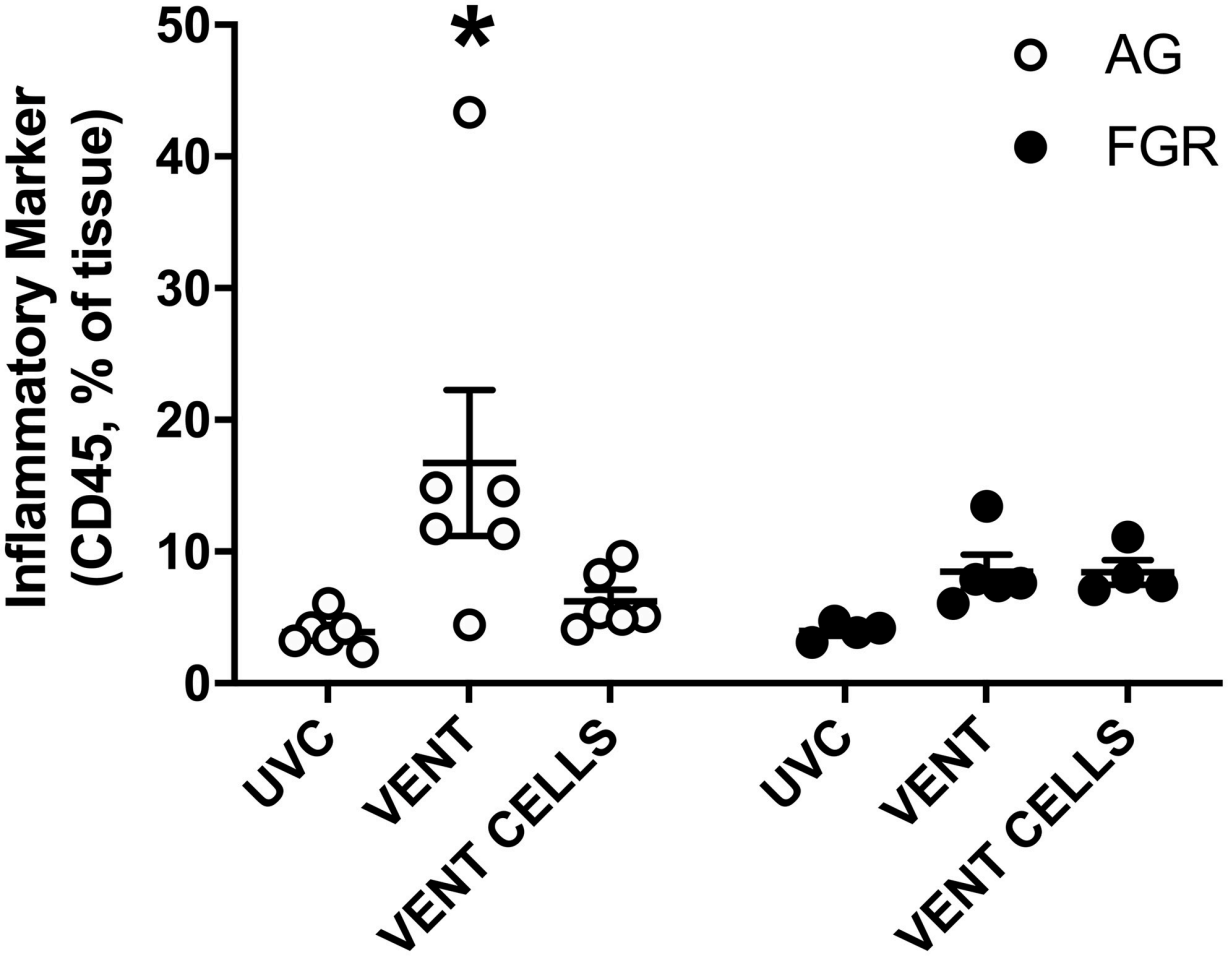
Inflammation. Data are mean ± SEM CD45 (inflammation marker) positive cells in appropriately grown (AG, white) and growth restricted (FGR, black) unventilated controls (AG_UVC_ and FGR_UVC_), following ventilation (AG_VENT_ and FGR_VENT_) and following ventilation and cell treatment (AG_CELLS_ and FGR_CELLS_). Data were compared using a two-way ANOVA. ^*^Indicates *p* < 0.05 treatment effects using a two-way ANOVA.

We used immunohistochemical analysis of CD45 to visually examine the infiltration of inflammatory cells into pulmonary tissue. Ventilation induced an inflammatory response in AG lambs (AG_UVC_ 3.9 ±.5 cells vs. AG_V_ 16.7 ± 5.5 cells, *P* = 0.0079). Inflammatory cell infiltration of the lungs was attenuated by treatment with UCB (Figure 5, *p* < 0.05) in AG lambs. Neither ventilation nor cell treatment induced a significant inflammatory response within the lungs of FGR lambs (FGR_UVC_ vs. FGR_V_ *p* = 0.5; FGR_V_ vs. FGR_CELLS_ *p* = 0.9).

**Figure 6:**
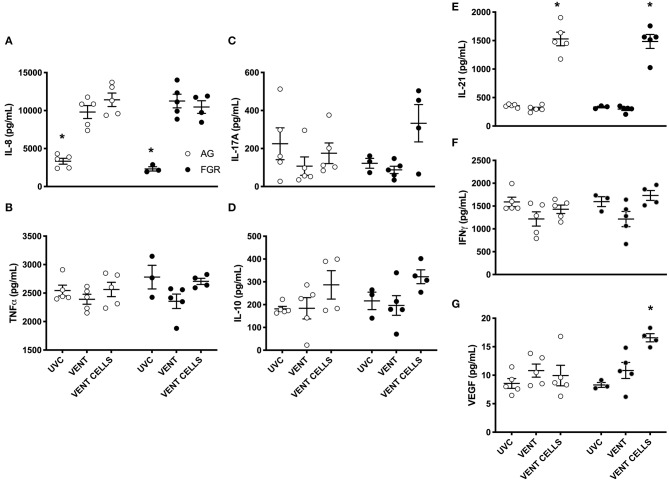
Inflammatory proteins. Data are mean ± SEM interleukin *(IL)-8* **(A)**, tumor necrosis factor alpha *(TNF*α*)* **(B)** *, IL-17A* **(C)**, *IL-10* **(D)**, *IL-21* **(E)** interferon gamma *(IFN)* **(F)**, and vascular endothelial growth factor *(VEGF)* **(G)**, cytokine levels in appropriately grown (AG, white) and growth restricted (FGR, black) unventilated controls (AG_UVC_ and FGR_UVC_), following ventilation (AG_VENT_ and FGR_VENT_) and following ventilation and cell treatment (AG_CELLS_ and FGR_CELLS_). ^*^Indicates *p* < 0.05 treatment effects using a two-way ANOVA.

A cytokine array was performed to further characterize the inflammatory profile within the lungs. Pro-inflammatory marker IL-8 was significantly increased in response to ventilation, in both AG and FGR lambs (*P* < 0.05), while treatment with UCB did not reduce IL-8 levels (Figure 6A). UCB treatment significantly increased IL-21 levels in FGR and AG lambs (Figure 6E). VEGF concentration was significantly increased in FGR lambs treated with UCB compared to both FGR_UVC_ (*P* = 0.007, Figure 6E) and FGR_V_ (*P* = 0.04) lambs. However, treatment with UCB cells significantly increase VEGF protein levels in FGR lambs compared to AG lambs treated with UCB cells (FGR_CELLS_ 16.6 ± 0.7 vs. AG_CELLS_ 9.9 ± 1.8, *P* = 0.002). There was no difference in TNFα, IL-17A, IL-10 or IFNγ levels between groups (Figures 6B–D,F, respectively).

## Discussion

Postnatally, FGR infants have increased risk of lung injury and bronchopulmonary dysplasia (BPD). Stem cell therapy has proven benefits to reduce VILI in preterm infants (34) as well as in reducing BPD incidence in preterm humans (33) and in animal models of neonatal lung injury (35). However, no previous studies have investigated if stem cell therapy is also beneficial for very low birthweight infants affected by growth restriction. In the current model, UCB therapy attenuated injury in AG but not FGR lambs. Our findings in appropriately grown lambs confirm previous studies showing improved lung structure following administration of placental stem-like cells, such as human amnion epithelial cells (34). Therefore, our current study increases the body of evidence for the use of UCB as an effective therapy for VILI in preterm infants who are appropriately grown. UCB treatment in FGR lambs increased pulmonary vascularization, but did not improve structural deficits in secondary septal crests, and increased collagen deposition, which is an early marker of fibrosis. Our research demonstrates that after 24 h of ventilation, UCB therapy shows differential effects in appropriately grown and growth restricted lambs, where protection from VILI was evident in AG lambs but not FGR lambs.

In the current study, we found little difference in the baseline lung morphology between the preterm unventilated FGR and AG fetuses, in line with previous observations from our group (4), although we induced early-onset placental insufficiency and FGR in this study, where we have previously examined late-onset (36). We hypothesized that longer exposure to placental insufficiency over a period of critical lung development would lead to the arrest of alveolar development as observed in other preclinical FGR studies (10, 11, 37). The latter being a probable mechanism for the increased risk of BPD (3) in this cohort. However, we did not see detectable differences in lung weights, when corrected for body weight, or baseline lung morphology in this study. Differences between the mode of inducing FGR and timing of compromise are most likely to contribute to differences in experimental outcomes. It is interesting to note that, despite a lack of gross or microscopic changes in lung morphology before ventilation, critical differences in response to ventilation were evident in the current study between AG and FGR lambs, suggesting sub-clinical alterations in lung development and/or biochemistry.

We have previously shown that AG and FGR newborns do not have significantly different ventilator requirements in the first 2 h of life (4, 38), however, in this study we extended these findings to show that, with a prolonged period of ventilation, FGR lambs begin to require a greater PIP to maintain V_T_, suggesting stiffer and less compliant lungs, a change that was sub-clinical throughout our experiment period as shown by dynamic compliance. All our lambs received antenatal betamethasone, which enhances surfactant production (39) and pulmonary function (40), and temporarily preserves lung compliance (41, 42) however, this may have had decreased efficacy in FGR. The rise in PIP over time may be a precursor to worsening VILI, lung compliance and ventilatory requirements, and certainly suggests that a longer period (>24 h) of study is necessary to tease apart differences associated with FGR.

Mechanical trauma as occurs with assisted ventilation induces an acute inflammatory response that initiates the inflammatory cascade and stimulation of inflammatory cytokine production (14, 16, 35). This was confirmed in the current study with ventilation significantly increasing pro-inflammatory cytokine IL-8 in FGR and AG lambs. In keeping with pulmonary IL-8 upregulation, infiltration of immune cells into the lungs (as evident by CD45^+^ expression) was also increased with ventilation, albeit this was statistically significant only in the AG lambs. Upregulation of inflammation following lung injury is well-described (4, 14, 16, 43, 44). Interestingly, IFNγ and TNFα were not altered in ventilated FGR or AG lambs. IFNγ is recognized as a key pro-inflammatory cytokine that has previously been shown to be up-regulated in response to lung injury (45). We did not see an up-regulation of IFNγ in the current study, this is likely due to the timing of lung collection, given that we measured inflammatory proteins in the lungs collected after 24 h of ventilation. IFNγ is seen to increase transiently in response to the initiation of ventilation with levels reducing over a period of hours-to-days after initial increase (46). TNFα is also found to be released in response to VILI in preterm neonates (45) however, pre-treatment with betamethasone, as was given in the current study, can prevent an increase in TNFα (47). UCB treatment in AG lambs attenuated immune cell infiltration into lungs but did not prevent the increase in IL-8 in either AG or FGR lambs. Moreover, UCB induced a 1.6-fold increase in lung IL-21 in AG and FGR lambs. IL-21 is a pro-inflammatory cytokine that promotes M2 “repair” to M1 “classically activated” macrophage phenotype, as well as increasing CD4^+^ and CD8^+^ T cell production (48), thus promoting inflammation. Persistence of inflammatory markers upregulated in response to mechanical ventilation in the current study are in contrast to previous reports where administration of placental stem cells increased expression of anti-inflammatory cytokines and reduced markers of lung inflammation following hyperoxic injury, thereby preventing downstream fibrosis and normalizing lung morphology (49). Differences between the findings here and those of previous studies for cell efficacy may reflect differences in the timing of tissue collection, mode of lung injury, the stage of lung development, or indeed the cells administered. It is perhaps too early to speculate whether the large increase in pulmonary IL-21 in response to UCB cells is a reparative or damaging effect, but it is increasingly well-understood that stem cells can modify a reparative response via immunomodulatory actions. This, however, is contingent on the inflammatory environment at the time of cell administration; stem cells introduced into a highly inflammatory host inhibit the protective capacity of stem cells (50), and can, in some instances, result in stem cells themselves becoming pro-inflammatory (51). This is a research area that requires further characterization, particularly for the vulnerable fragile preterm lungs.

Consistent with previous findings, we observed suppression of septal crests density following ventilation (16), and elastin distribution became diffuse along the alveolar wall; in AG lambs elastin density was significantly reduced and a similar (non-significant) trend was seen in lungs from FGR lambs. Ventilation in neonatal sheep and mice induces an upregulation of elastin production, but not the regulators of elastin assembly, leading to disordered accumulation of elastin along the alveolar walls (52). The qualitative changes we observed are likely a precursor to abnormal elastin deposition, highlighting the importance of treating in this acute period, before structural changes. Collagen, elastin and α-smooth muscle actin are essential structural components of the lung (53), and perturbations to the density and distribution of these factors will alter the mechanics of the lung. Twenty-four hours of ventilation in our preterm lambs decreased α-smooth muscle actin density in the FGR cohort compared to unventilated controls. Long-term (1 month) of ventilation increases α-smooth muscle actin (54), and thus we may have observed a transient decrease in this study, before a secondary compensatory increase. The mechanisms underlying the decreased α-smooth muscle actin in this current study are unknown, however, it is interesting to speculate on the possible role of nitric oxide (NO). In culture, increased NO reduces smooth muscle production, whilst inhibition of NO results in smooth muscle accumulation (55). It is well-accepted that growth restriction impairs NO handling (56, 57), and we have shown decreased content and altered distribution of the NO precursor, eNOS, following 2 h of ventilation (58). However, exposure to hyperoxia in the first day of life may increase local NO production due to impaired NO handling in FGR newborns, thereby resulting in inhibition of smooth muscle production.

Septal crest density is vital for increasing the surface area available for gas exchange. Ventilation induced a decrease in septal crest density in AG and FGR lambs, which is representative of simplification of the airways, and this is a hallmark of bronchopulmonary dysplasia (59). UCB was protective for septal crest density in the lungs of AG lambs, but not the FGR lambs. Despite this, we observed an improvement of injury heterogeneity in both AG and FGR lambs with treatment. Therefore, UCB may also promote, via paracrine mechanisms, surfactant production to reduce atelectasis.

Strikingly, there was an increase in the collagen to tissue ratio after UCB administration in FGR lambs. In previous studies, UCB-derived mesenchymal stem cells (MSCs) have increased fibroblast formation compared to those sourced from adipose tissue or bone marrow (13, 60). Despite this, no previous studies observed increased collagen; and UCB-MSCs or mononuclear cells administered to mice with VILI did not alter levels of TGF-β, a regulator of collagen production, or collagen content 14 days after cell administration (15, 50). However, to the authors' knowledge, there are no other studies specifically aimed at determining the efficacy of cell treatment in a growth restricted population. Whilst it is possible that the increase in collagen in FGR lambs in the current study is transient, given the relationship between collagen deposition and fibrotic disease, this relationship requires additional research.

Alveolar epithelial cells are a key source of increased cell proliferation following ventilation induced lung injury (61). Interestingly, cell proliferation was significantly increased in ventilated AG lambs but reduced in FGR ventilated lambs. FGR is linked with lower levels of growth factors and decreased pulmonary cell growth in culture (11). We have previously shown that glucocorticoids reduce cell proliferation, both in AG and FGR fetuses (38) but since all groups received betamethasone, this is unlikely to have caused the difference observed here. It is more likely that the growth restricted lung does not respond to stretch by inducing proliferation, a well-established mechanism in the lungs of AG infants. Indeed, another key stretch response, the baroreflex response, is significantly attenuated in growth restricted fetuses (62), suggesting a possible decreased responsivity to this critical form of stimulus. Overall, these unexpected findings re-emphasize that even though ventilator requirements and fetal histology was not different between groups, FGR lungs respond differently to ventilation compared to AG lungs, and these changes may underpin the increased vulnerability to injury and long-term morbidity in FGR offspring. UCB treatment did not improve cell proliferation in FGR lungs. The underlying physiology is unknown, however investigating which cells are proliferative in AG would be of interest.

Treatment with UCB in FGR ventilated lambs promoted blood vessel growth as evidenced by the increased VEGF and vessel number, a finding not seen in AG lambs. VEGF is a potent inducer of angiogenesis and decreased VEGF expression is seen in newborns with BPD. *In vitro*, both MSCs (63) and endothelial progenitor cells (64) promote angiogenesis, via increases in VEGF. It is possible that, given time, increased vascularization of the lung would promote restoration of alveolarization, given the known positive relationship between these two factors (65). It is interesting, but not immediately apparent, why VEGF was increased in FGR, but not AG ventilated lambs. It is known that hypoxia increases VEGF production, and placental insufficiency directly exposes the developing fetus to chronic hypoxemia, which may in turn, result in impaired or altered hypoxia sensing and handling, and response.

How stem cells exert a reparative benefit is still not fully understood, however several mechanisms are possible. They may migrate to areas of injury and release trophic factors to reduce inflammation and promote endogenous repair mechanisms or they may alter systemic immune-modulatory responses (66). We observed only small numbers of UCB cells within the lungs, suggesting that their main effect was not via cell engraftment, but rather a paracrine effect as expected. *In vitro*, MSCs demonstrated preferential migration to hyperoxia-injured lung tissue rather than control medium or healthy lung tissue (49), suggesting stem cells are specifically recruited to sites of injury. Intra-tracheal UCB administration provides direct access to the lung and may improve lung outcomes (35), however, given that intubation is increasingly infrequent in pediatrics (67), systemic administration of cells is more clinically relevant.

It is now well-established that a poor uterine environment has the potential to program disease in later life (68). Emerging evidence also suggests that subtle, sub-clinical alterations are present in the lung (69) at the time of birth, which not only changes the function of the organ but can also alter the response of the organs to additional insults. It is likely that the lung is similarly affected, where an altered response to injury in FGR as compared to AG offspring has been demonstrated in animal models (70) and humans (3). It follows that treatments also may have different therapeutic ranges in infants following a sub-optimal pregnancy, and therefore further research is required to determine how to best target therapies to this population.

### Limitations

We administered the UCB cells at a dose of 25 million cells/kg, based on evidence that this level is neuroprotective, and gave this dose 1 h after birth. It is possible that this cell dose and timing is not optimal for treatment of the lungs of the FGR infant and highlights the need to consider the FGR population independent of AG preterm infants. As with many other therapeutics, the timing of stem cell administration is vital, and it is possible that delaying cell administration until after the primary inflammatory phase is more protective in the lung, as has been observed *in vitro* (51). Further studies should examine this possibility and also determine the effects of UCB on VILI, in AG and FGR animals, beyond the 24-h. This will offer a better understanding of how UCB may benefit functional and morphological outcomes and chronic lung disease. Finally, the FGR infant has multi-organ dysfunctions and, while we only examined lung outcomes in the current study, optimizing postnatal therapeutics for multiple organs, including the lung, brain and cardiovascular system, must be considered. Treatment strategies to improve neurological structure and function are now being examined, including cell therapies. The current study suggests that we need additional targeted research to determine the interaction between organ systems, possible developmental programming and postnatal treatments, such as cell therapies.

### Conclusion

FGR newborns have an increased risk of bronchopulmonary dysplasia and, whilst there is currently no cure for FGR or lung disease in this vulnerable cohort, UCB stem cells have shown potential therapeutic benefits in preterm infants. Here we sought to determine if UCB cells would also be beneficial for growth restricted preterm newborns. Our results have demonstrated that UCB shows promising anti-inflammatory benefits for treating ventilation induced lung injury in appropriately grown newborn lambs. However, UCB treatment was not equally effective for FGR infants, where it promoted angiogenesis, did not reverse detrimental changes in lung structure, and increased collagen and its precursor αSMA, which may be injurious. Interestingly, the pulmonary response to cell administration was differentially regulated in AG and FGR lambs, wherein UCB increased VEGF and decreased cell proliferation in FGR lambs only, however, whether this would be beneficial or not for the future of the offspring is yet to be determined. Our study is the first to highlight that the FGR infant responds differently to cell therapy, and these results suggest that developmental programming *in utero* needs to be considered when giving postnatal treatments.

## Data Availability Statement

All datasets generated for this study are included in article/Supplementary Material.

## Ethics Statement

Ethical approval for all experimental procedures utilized in this project was granted through the Monash Medical Centre Animal Ethics Committee (approval number MMCA2014-04).

## Author Contributions

BA, AM, GP, and SM conceived and designed the analysis. BA, HY, AM, CM, MC-M, YP, AS, GJ, GP, and SM collected and contributed to the data. BA and HY performed the analysis and BA, HY, AM, CM, MC-M, YP, AS, GJ, GP, and SM wrote the paper.

### Conflict of Interest

The authors declare that the research was conducted in the absence of any commercial or financial relationships that could be construed as a potential conflict of interest.
