# Supplementary figures and images for: Is Umbilical Cord Blood Therapy an Effective Treatment for Early Lung Injury in Growth Restriction?
Source: Front Endocrinol (Lausanne). 2020 Mar 3;11:86. doi: 10.3389/fendo.2020.00086 (PMC7063054; doi:10.3389/fendo.2020.00086)

## Slide 1
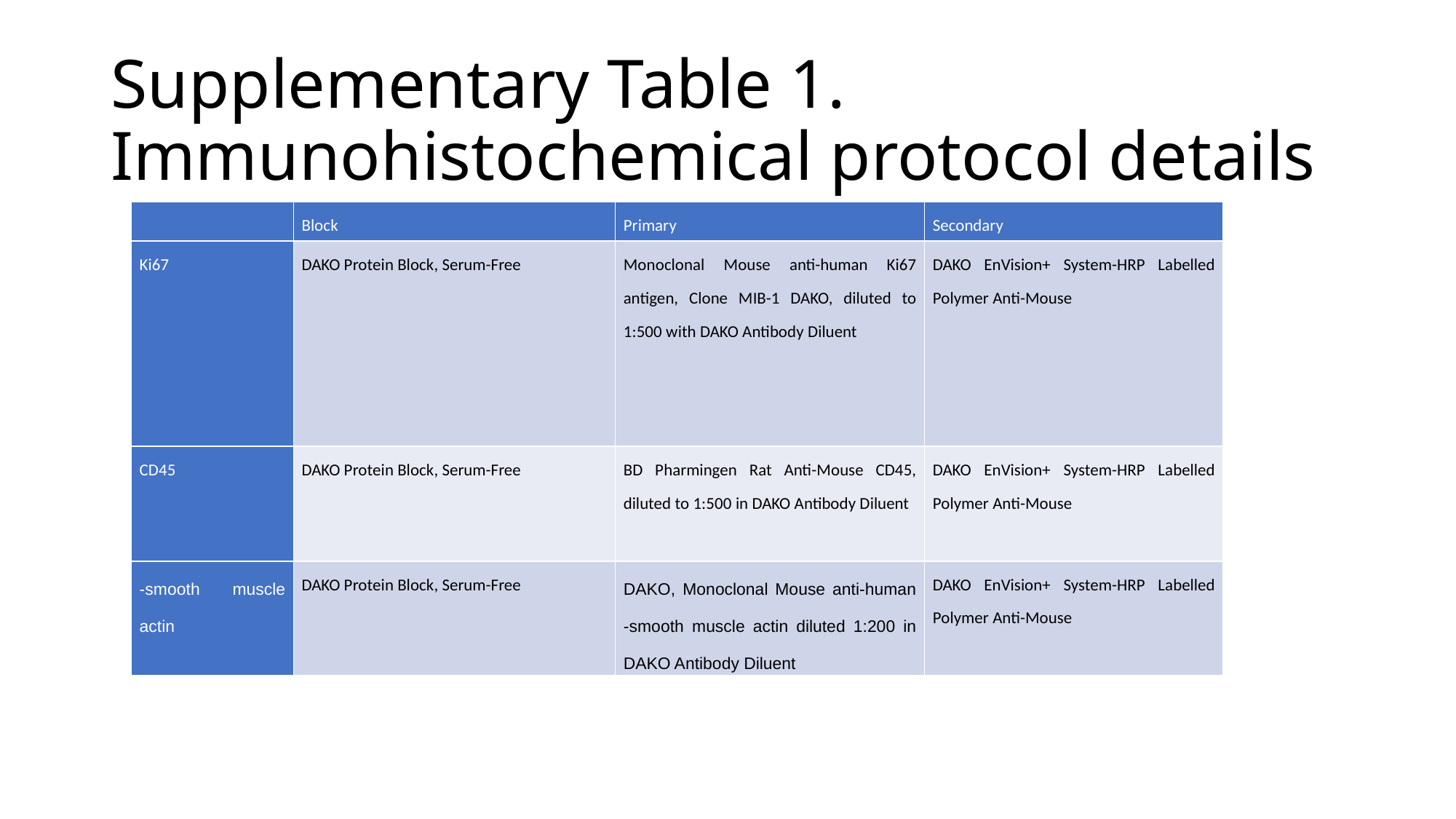

# Supplementary Table 1. Immunohistochemical protocol details

Supplement: Supplementary file 1 [file Presentation_1.pptx]
